# Experience and Reflection From Inpatient Staff at an Intellectual Disability Hospital During COVID-19

**DOI:** 10.1192/bjo.2022.172

**Published:** 2022-06-20

**Authors:** Rahul Malhotra, Zeenish Azhar

**Affiliations:** Betsi Cadwaladr University Health Board, Bangor, United Kingdom

## Abstract

**Aims:**

The COVID-19 pandemic and the associated impact on the NHS led to particular challenges for Intellectual Disability (ID) inpatient hospitals across the country. The aim of this Research Project is to gather the experience of Inpatient staff in our local ID Hospital following the first wave of COVID-19 pandemic in July 2020.

**Methods:**

We gathered data by means of survey from inpatient staff including ‘staff nurses’ and ‘health care support workers’ from 2 cute assessment and treatment units and 1 rehabilitation unit over the preceding 3 months. We obtained 15 responses. We gathered quantitative data via a questionnaire on the views of staff regarding the service provision for patients and staff during COVID-19. We also gathered qualitative data on learning points and how things would have been done differently in hindsight.

**Results:**

The responses were anonymised, directly transcribed, coded and grouped into themes. 67% of staff stated appropriate type and quantity of Personal Protective Equipment was available. 60% of staff stated it was ‘easy’ to access a General Practitioner for patient reviews. 60% of staff stated, there was a change in arrangements for Do Not Resuscitate/Escalation plans during COVID-19. 47% of staff stated there was availability of virtual or face-to-face clinical training support. 67% of staff did not take sickness leave due to symptoms or contact with a COVID-19 patient. 67% of staff did not receive or found it difficult to access a COVID-19 test. 47% of staff reported a negative impact of the pandemic on their physical and mental health well being. 13% of staff found the Counselling/emotional Support helpful.

Some of the key themes that emerged in the qualitative data analysis included the importance of being grateful for personal health and well being, move lives could be saved if earlier and more frequent testing was available during the first wave, delays in the arrival of PPE in the hospital and ideas to mitigate risk by designating members of staff to a fixed work area to reduce mixing.

**Conclusion:**

A wide range of reflections, suggestions and feedback were obtained during the research project which will be helpful to plan and organise services moving forward should future waves of COVID-19 emerge.

